# Fungal His-Tagged Nitrilase from *Gibberella intermedia*: Gene Cloning, Heterologous Expression and Biochemical Properties

**DOI:** 10.1371/journal.pone.0050622

**Published:** 2012-11-30

**Authors:** Jin-Song Gong, Heng Li, Xiao-Yan Zhu, Zhen-Ming Lu, Yan Wu, Jing-Song Shi, Zheng-Hong Xu

**Affiliations:** 1 Laboratory of Pharmaceutical Engineering, School of Medicine and Pharmaceutics, Jiangnan University, Wuxi, People's Republic of China; 2 The Key Laboratory of Industrial Biotechnology, Ministry of Education, Jiangnan University, Wuxi, People's Republic of China; 3 Laboratory of Bioactive Products Processing Engineering, School of Medicine and Pharmaceutics, Jiangnan University, Wuxi, People's Republic of China; Soonchunhyang University, Korea, Republic of

## Abstract

**Background:**

Nitrilase is an important member of the nitrilase superfamiliy. It has attracted substantial interest from academia and industry for its function of converting nitriles directly into the corresponding carboxylic acids in recent years. Thus nitrilase has played a crucial role in production of commercial carboxylic acids in chemical industry and detoxification of nitrile-contaminated wastes. However, conventional studies mainly focused on the bacterial nitrilase and the potential of fungal nitrilase has been far from being fully explored. Research on fungal nitrilase gene expression will advance our understanding for its biological function of fungal nitrilase in nitrile hydrolysis.

**Methodology/Principal Findings:**

A fungal nitrilase gene from *Gibberella intermedia* was cloned through reverse transcription-PCR. The open reading frame consisted of 963 bp and potentially encoded a protein of 320 amino acid residues with a theoretical molecular mass of 35.94 kDa. Furthermore, the catalytic triad (Glu-45, Lys-127, and Cys-162) was proposed and confirmed by site-directed mutagenesis. The encoding gene was expressed in *Escherichia coli* Rosetta-gami (DE3) and the recombinant protein with His_6_-tag was purified to electrophoretic homogeneity. The purified enzyme exhibited optimal activity at 45°C and pH 7.8. This nitrilase was specific towards aliphatic and aromatic nitriles. The kinetic parameters *V*_max_ and *K*_m_ for 3-cyanopyridine were determined to be 0.81 µmol/min·mg and 12.11 mM through Hanes-Woolf plot, respectively. 3-Cyanopyridine (100 mM) could be thoroughly hydrolyzed into nicotinic acid within 10 min using the recombinant strain with the release of about 3% nicotinamide and no substrate was detected.

**Conclusions/Significance:**

In the present study, a fungal nitrilase was cloned from the cDNA sequence of *G. intermedia* and successfully expressed in *E. coli* Rosetta-gami (DE3). The recombinant strain displayed good 3-cyanopyridine degradation efficiency and wide substrate spectrum. This fungal nitrilase might be a potential candidate for industrial applications in carboxylic acids production.

## Introduction

Recently, biocatalysis has been widely explored to improve the sustainability and efficiency for industrial production of fine chemicals, which are used as intermediates for pharmaceuticals, agrochemicals, materials and food ingredients [1], [2]. The applications mainly lie in the exploration of the attractive features owned by these biocatalysts with respect to substrate selectivity, chemoselectivity and catalysis at ambient temperatures and pressures [2]. Several enzymes have been successfully employed in the biocatalytic reactions to prepare industrial commodities [3].

As an valuable biocatalyst, nitrilase (EC 3.5.5.1) are used for enzymatic transformation of nitriles directly to corresponding carboxylic acids with releasing ammonia [4]. Nitrilase-mediated biocatalysis has attracted substantial interests owning to its crucial importance for production of carboxylic acids in chemical industry and detoxification of nitrile-contaminated wastes [5], [6], [7]. Within the past few decades, a considerable amount of works have focused on various aspects about nitrilases. The first nitrilase was discovered from barley and described by Thimann and his co-workers [8]. So far, several nitrilases of different origins have been obtained and investigated in depth. Nitrilases have been mainly found in bacteria belonging to genera *Bacillus*, *Klebsiella*, *Alcaligenes*, *Acinetobacter*, *Nocardia*, *Pseudomonas*, and *Rhodococcus* [7], fungi belonging to genera *Fusarium* and *Aspergillus* [9], [10], plants belonging to genera *Arabidopsis*, *Brassica*, *Lupinus*, and *Nicotiana* [11], and animals [12]. With regard to catalytic mechanism of nitrilase, it was speculated that the carbon atom of the cyano group in nitrile molecule was attacked by a thiol group in cysteine residue of the catalytic triad, forming a tetrahedral thiomidate intermediate. And then it was attacked by two water molecules and protonation of the nitrogen atom, associating with the release of ammonia [13].

The first gene encoding nitrilase was cloned from *K. ozaenae* and expressed in *Escherichia coli* [14]. Some other recently expressed or characterized nitrilases are known from *A. faecalis*, *R. rhodochrous*, *Alcaligenes* sp., *P. putida*, and so on [15], [16], [17], [18]. When referring to the fungal nitrilase, it was proved to exhibit obvious advantages over most bacterial nitrilase in terms of stability and catalytic activity [19]. It is considered to be a kind of useful industrial enzyme resource and play a crucial role in potential commercial production. However, to date, only a fungal nitrilase was cloned from a wild–type *A. niger* K10 in a recent literature [20]. On the other hand, the genome mining was performed to search fungal nitrilase from putative protein in GenBank, and the catalytic activity of hypothetical nitrilases from *A. niger*, *Gibberella moniliformis* and *Neurospora crassa* was confirmed through their synthetic genes being expressed in *E. coli* [21], [22]. Moreover, two nitrilases from *Bradyrhizobium japonicum* and *Pyrococcus abyssi*, which were specific for mandelonitrile, were also cloned via rational genome mining, respectively [23], [24]. On the whole, most of the reported works have mainly focused on bacterial nitrilase and the potential of fungal nitrilase has been far from being fully explored. The reason mainly lies in the limited availability of fungal nitrilase gene.

In our previous work, a fungus of *G. intermedia* CA3-1 was isolated from soil in our laboratory and proved to be promising for potential applications in industrial production of carboxylic acids from nitriles. And it was deposited in the China General Microbiological Culture Collection Center (CGMCC 4903; Beijing, China). In the present study, the fungal nitrilase from *G. intermedia* CA3-1 was successfully cloned through reverse transcription-PCR (RT-PCR) and heterologously expressed in *E. coli*. The catalytic triad of fungal nitrilase was proposed and relevant residues were confirmed by constructing nitrilase variants through overlap extension PCR (OE-PCR). Furthermore, the recombinant nitrilase was purified and the biochemical properties of the purified enzyme were studied. This study would improve our understanding for the biological function of fungal nitrilase in nitrile hydrolysis. Furthermore, the recombinant expression could lay the foundation for future works on molecular modification of fungal nitrilase gene and large-scale preparation of this recombinant fungal nitrilase.

## Results and Discussion

### Cloning and sequence analysis of the fungal nitrilase gene

**Figure 1:**
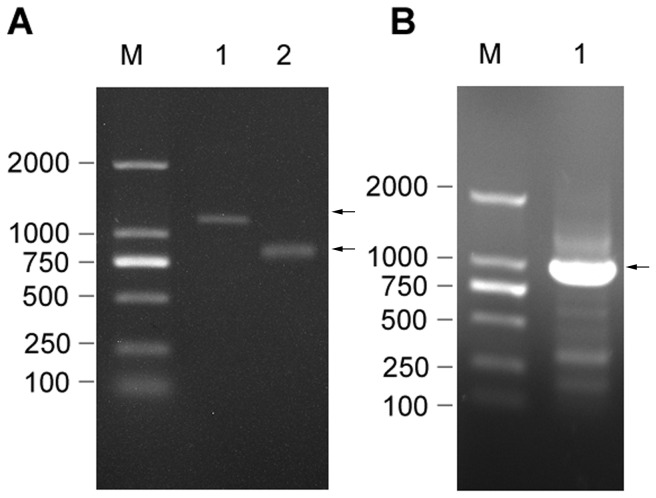
Cloning of the fungal nitrilase gene from (A) Amplification of the cDNA sequence from RNA through RT-PCR. M: DL2000 marker; Lane 1: PCR product was amplified using primers am and M13 Primer M4; Lane 2: PCR product was amplified using primers bm and M13 Primer M4. (B) Amplification of the DNA sequence encoding nitrilase gene. M: DL2000 marker; Lane 1: PCR product was amplified using primers P1 and P2.

The fungal nitrilase gene was amplified from *G. intermedia* CA3-1 through RT-PCR. A 1.1 kb cDNA fragment was obtained as the major product using primers am and M13 Primer M4, as shown in Figure 1A. This amplification result was further confirmed by nested-PCR using bm and M13 Primer M4 as primers, which showed that a 0.9 kb fragment was obtained with the above 1.1 kb fragment after being purified as the template. The encoding gene of fungal nitrilase was amplified from cDNA sequence with primers P1 and P2. PCR product was subjected to agarose gel electrophoresis (Figure 1B) and it was revealed that a fragment of about 1.0 kb was generated. This fragment was purified and ligated with pMD19-T, and then the recombinant plasmid pMD19T-Nit was transformed into *E. coli* DH5α competent cells by heat shock. Positive clones with ampicillin resistance were singled out and sequenced.

**Figure 2:**
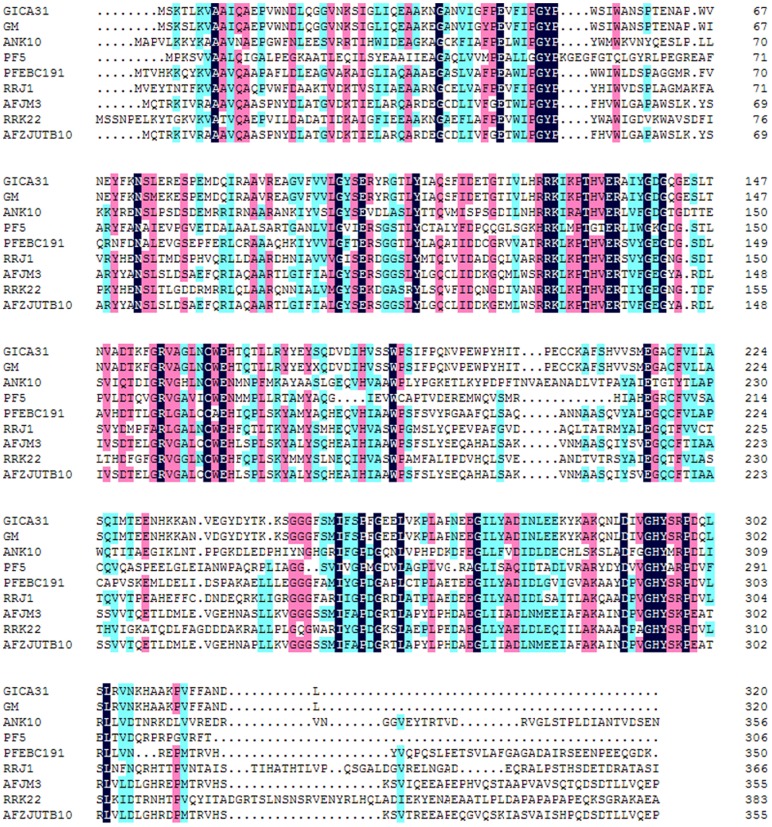
Amino acid sequence alignment of nitrilases from different origins. GICA31, the nitrilase from *G. intermedia* CA3-1; GM, the putative nitrilase from *G. moniliformis* (ABF83489); ANK10, the nitrilase from *A. niger* strain K10 (ABX75546); PF5, the nitrilase from *P. fluorescens* Pf-5 (YP_260015); PFEBC191, the nitrilase from *P. fluorescens* EBC191 (AAW79573); RRJ1, the nitrilase from *R. rhodochrous* J1 (BAA01994); AFJM3, the nitrilase from *A. faecalis* JM3 (BAA02684); RRK22, the nitrilase from *R. rhodochrous* K22 (BAA02127); AFZJUTB10, the nitrilase from *A. faecalis* ZJUTB10 (AEP34036).

The sequence analysis demonstrated that the target fragment contained an open reading frame of 963 bp in length which encoded a protein of 320-amino-acid with a theoretical molecular mass of 35.94 kDa. The sequence alignment results (Figure 2) indicated that the fungal nitrilase from *G. intermedia* CA3-1 showed 97% identity with the putative nitrilase from *G. moniliformis* (ABF83489). The identities of *G. intermedia* CA3-1 nitrilase gene to the other nitrilases from *A. niger* (ABX75546), *P. fluorescens* (YP_260015), *P. fluorescens* (AAW79573), *R. rhodochrous* (BAA01994), *A. faecalis* (BAA02684), *R. rhodochrous* (BAA02127) and *A. faecalis* (AEP34036) were 34%, 27%, 39%, 38%, 36%, 33% and 36%, respectively.

### Heterologous expression of *Gibberella* nitrilase and its purification

To express the *Gibberella* nitrilase encoding gene, the double-digested pMD19T-Nit and pET-28a(+) were ligated with T4 DNA ligase, and the recombinant expression plasmid pET28a(+)-Nit was constructed. It was transformed into competent cells of *E. coli* Rosetta-gami (DE3). The recombinant strain of *E. coli* Rosetta-gami (DE3)/pET28a(+)-Nit was induced by IPTG. It was observed that the recombinant strain harboring nitrilase could hydrolyze 3-cyanopyridine with an activity of 0.5 U/mg dry cell without further optimization. In another control experiment which expressed the plasmid pET-28a(+) in *E. coli* Rosetta-gami (DE3), no nitrilase activity was detected.

When the resting cells of recombinant strain were applied in the nitrile hydrolysis reaction, 100 mM 3-cyanopyridine (10.4 g/L) was fully converted within 10 min. And the corresponding product nicotinic acid was obtained at the same time while nicotinamide was by-produced with about 3% of the total product. The volumetric productivity (g nicotinic acid/L·h) and the catalyst productivity (g nicotinic acid/g dry cell) were calculated to be 69.0 and 1.53, respectively. The formation of amide has also been reported in some other nitrilase-producing strains especially filamentous fungi and it could be overcome by using the cascade reaction of nitrilase-amidase [25]. The half-life of the resting cells at 30, 40 and 50°C was determined to be 24.75, 2.55 and 1.34 h, respectively. It indicated that this recombinant nitrilase exhibited a moderate thermostability. This made the performance of fed-batch reaction possible for continuous production of carboxylic acid from nitrile conversion in practical application.

**Figure 3:**
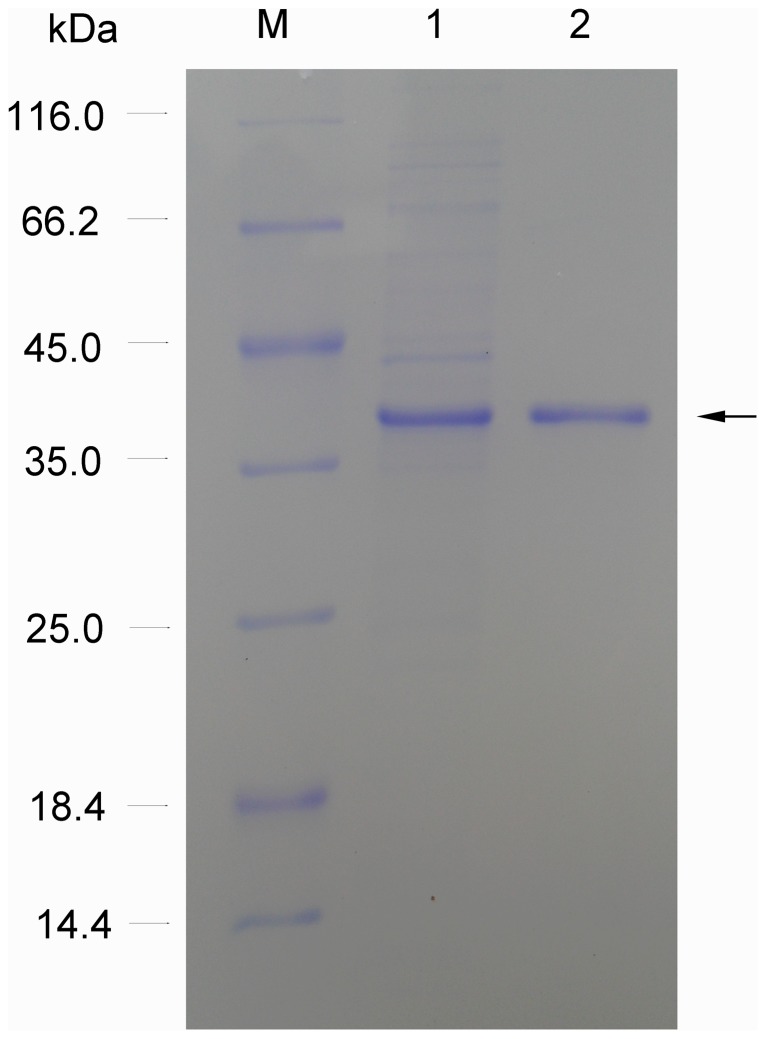
SDS-PAGE analysis of the recombinant nitrilase. M: protein standard marker; Lane 1: the supernatant of the cell-free extract; Lane 2: purified nitrilase by Ni-NTA column.

The recombinant nitrilase was purified using a Ni-NTA column. As can be seen from the SDS-PAGE analysis in Figure 3, the protein has been purified to electrophoretic homogeneity. The molecular mass of recombinant nitrilase was approximately 37.0 kDa, as determined by SDS-PAGE.

### Site-directed mutagenesis

The structure prediction of *G. intermedia* nitrilase using http://swissmodel.expasy.org/ programs demonstrated that this nitrilase exhibited moderate similarity with the crystal structure of NAD^+^ synthetase from *Streptomyces avermitilis* (3n05A), which belongs to the nitrilase superfamily. As previously reported, nitrilase contained the invariant catalytic triad residues, glutamate, lysine and cysteine as one of the members in the nitrilase superfamily [26]. The amino acid residues Glu-45, Lys-127, and Cys-162 were found to be highly conserved and proposed to be the catalytic triad. The positions of these proposed residues were confirmed by construction of the nitrilase variants with mutation at the relevant positions through OE-PCR. The mutants were verified by sequencing, heterologous expression and conversion of 3-cyanopyridine. The nitrilase assay results of constructs showed that the mutants Glu45Gln, Lys127Arg and Cys162Gly were completely devoid of nitrilase activity using 3-cyanopyridine as the substrate and no activity could be detected in the reaction mixture. The crystal structure of *G. intermedia* nitrilase should be determined to pave the way for further molecular modification.

### Effects of environmental factors on nitrilase activity

**Figure 4:**
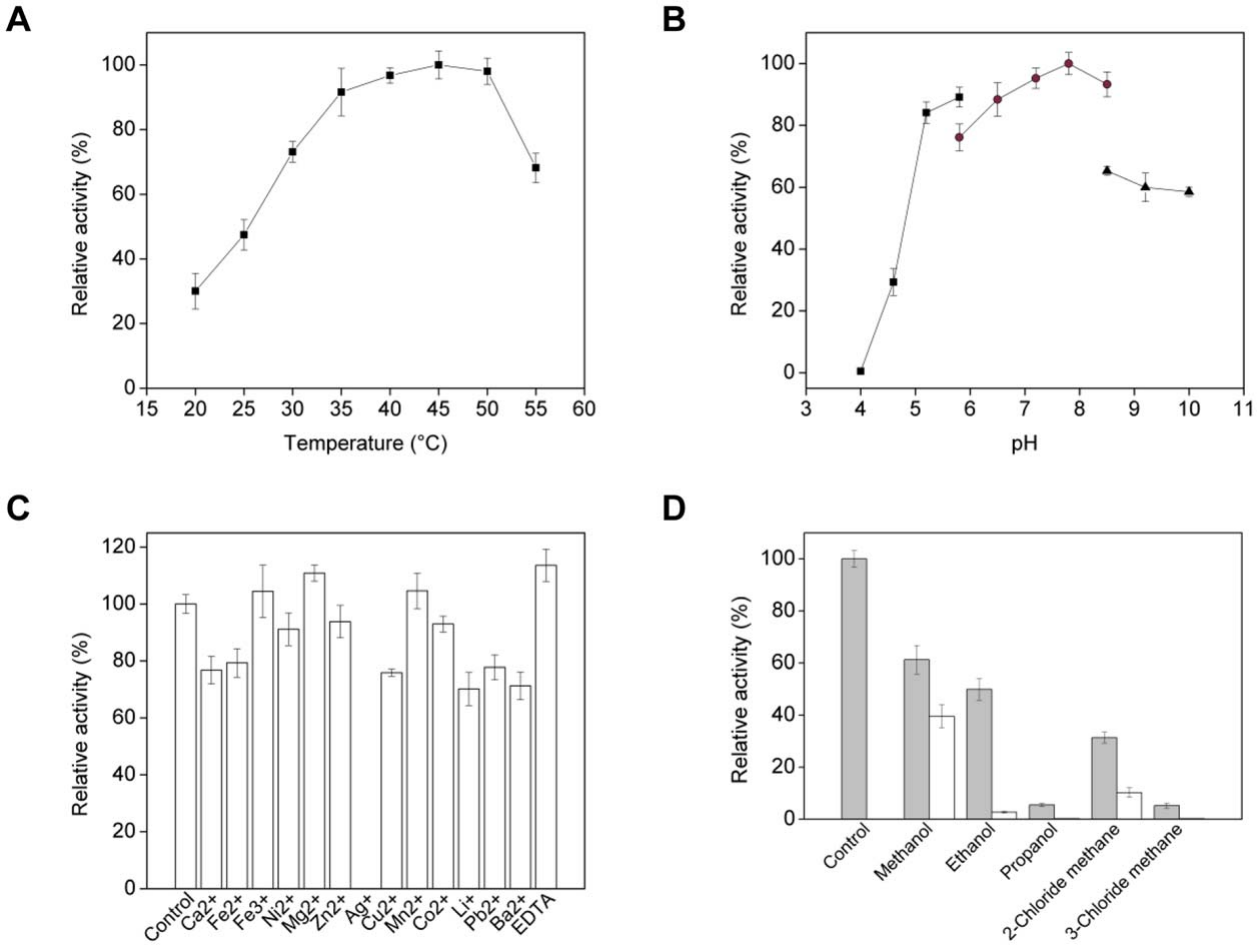
Effects of environmental factors on the activity of nitrilase. (A) Effects of temperature on nitrilase activity. The assay is based on ammonia generated in the reaction mixture. The relative activity was expressed at the percentage of the activity at 45°C (specific activity 3.7 U/mg protein). (B) Effects of pH on nitrilase activity. The relative activity was expressed at the percentage of the activity at pH 7.8 (specific activity 2.9 U/mg). Symbols: Sodium citrate buffer (▪); sodium phosphate buffer (•); tris-HCl buffer (▴). (C) Effects of metal ions on enzyme activity. Appropriate metal ion solution was added to the reaction mixture at a final ion concentration of 1 mM, and the relative activity was expressed at the percentage of the activity without addition of metal ions (specific activity 2.8 U/mg). (D) Effects of organic solvents on enzyme activity. The relative activity was expressed at the percentage of the activity without addition of organic solvents (specific activity 2.8 U/mg). Symbols: 5% (v/v) organic solvent (▪); and 20% (v/v) organic solvent (□).

The highest activity of the *Gibberella* nitrilase was found at the temperature of 45°C (Figure 4A). The nitrilase activity gradually increased from 20°C to 45°C and lost rapidly above 50°C, which were similar to those from *R. rhodochrous* J1 [27], *F. solani* IMI196840 [19] and *A. faecalis* JM3 [28]. The *Gibberella* nitrilase showed optimum activity at pH 7.8, which was similar to the two *F. solani* nitrilases [19], [29]. As shown in Figure 4B, this enzyme exhibited a broad pH range from pH 6–10, which was comparable to that of nitrilase from *F. oxysporum* f sp. *melonis* (pH optimum of 6–11) [30]. Interestingly, there is a certain difference between wild and recombinant enzyme for the optimum conditions (the work on purified nitrilase of wild-type is undergoing, data not shown). It may contribute to the His-tag carried in the recombinant nitrilase and some acceptable changes of enzyme structure after the fungal nitrilase expressed in *E. coli*.

The nitrilase activity in presence of metal ions and EDTA was determined (Figure 4C). The enzyme activity of *G. intermedia* CA3-1 was strongly suppressed by thiol binding reagents Ag^+^, like those from *P. abyssi* [24], *F. solani* O1 [29] and *A. niger* K10 [31]. This phenomenon indicated that thiol group was indispensable for the catalytic activity of nitrilase, which was in good agreement with the catalytic mechanism of nitrilase, as described above. On the other hand, Fe^3+^, Mg^2+^ and Mn^2+^ were proved to be favorable for the nitrilase activity, and those ions increased enzyme activity to 104%, 110% and 105%, respectively. Furthermore, the metal chelating agent, EDTA, could improve the nitrilase activity by 14%. Some other ions, such as Ni^2+^, Zn^2+^ and Co^2+^ softly decreased the enzyme activity. This was nearly similar to the nitrilase from *Alcaligenes* sp. ECU0401 [16].

Due to the poor solubility of substrates (nitriles) or products (carboxylic acids) in the bioconversion reaction, nitrilase-mediated conversion reaction in organic solvents was evaluated to determine the degree of resistance to organic solvents for nitrilase. As shown in Figure 4D, the enzyme was relatively active in 5% (v/v) methanol with 60% of the enzyme activity remained. Relative activity of 49%, 6%, 31% and 5% were detected in 5% (v/v) of ethanol, propanol, 2-chloride methane, and 3-chloride methane, respectively. When 20% (v/v) of organic solvent was used, the enzyme activity sharply decreased and only could detect very low activity except that in methanol and 2-chloride methane (40% and 10% of activity retained, respectively). Moreover, no activity was found with addition of butanol and acetic ether (data not listed).

### Substrate spectrum of the recombinant nitrilase

The recombinant nitrilase from *G. intermedia* CA3-1 could be able to hydrolyze various nitriles, as can be seen from Table 1. Impressively, this nitrilase was highly specific towards heterocyclic nitriles such as 3- and 4-cyanopyridine, similar to the nitrilase from *A. niger* K10 [31]. Furthermore, high activities were observed for aliphatic nitriles including valeronitrile, isovaleronitrile, acrylonitrile and dinitriles such as adiponitrile, succinonitrile and iminodiacetonitrile. On the other hand, appropriate activities were found for aromatic nitriles, benzonitrile and phenylacetonitrile (Table 1). He et al. recently reported the resting cells of *Rhodococcus* sp. CCZU10-1 also harbored excellent nitrilase activity for aliphatic and aromatic nitriles [4]. However, no activity was detected when mandelonitrile, the intermediate for mandelic acid production, was used as substrate of the present nitrilase.

**Table 1:** Substrate spectrum of the recombinant nitrilase from *G. intermedia* CA3-1.

Substrate	Relative activity (%)
2-Cyanopyridine	0.07±0.02
3-Cyanopyridine	100.00±5.56
4-Cyanopyridine	69.25±1.93
2-Chloro-3-cyanopyridine	0.21±0.05
2-Chloro-4-cyanopyridine	7.48±0.89
Benzonitrile	42.14±4.32
Phenylacetonitrile	21.59±1.12
4-Methoxy phenylacetonitrile	0.49±0.04
Mandelonitrile	ND
Glycinonitrile	23.27±0.98
Acetonitrile	2.31±0.07
Acrylonitrile	43.96±3.79
Valeronitrile	45.07±3.21
Isovaleronitrile	47.17±2.59
Succinonitrile	64.22±5.17
Geranyl nitrile	ND
Adiponitrile	106.29±4.28
Iminodiacetonitrile	58.07±1.91

Nitrilase activity was determined under the standard assay conditions. It was based on ammonia generated in the reaction mixture. The specific activity with 3-cyanopyridine was set at 100% (specific activity 2.8 U/mg protein).

ND means not detected.

### Enzymatic kinetics

**Figure 5:**
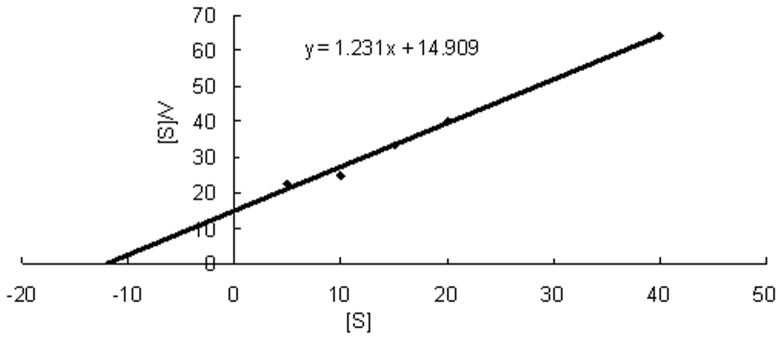
Hanes-Woolf plot of the purified nitrilase.

As shown in Figure 5, kinetic parameters of the recombinant nitrilase were determined with 3-cyanopyridine as the substrate in the present study. It was calculated that *V*_max_ and *K*_m_ were 0.81 µmol/min·mg and 12.11 mM through Hanes-Woolf plot, respectively. These values were similar to that of nitrilase from *R. rhodochrous* NCIMB 11216 for benzonitrile, which had a *K*_m_ of 8.83 mM and a *V*_max_ of 0.57 µmol/min·mg protein [32].

## Conclusions

A gene encoding a protein with fungal nitrilase activity was cloned from the cDNA sequence of *G. intermedia* CA3-1 and successfully expressed in *E. coli* Rosetta-gami (DE3). Site-directed mutagenesis confirmed that Glu-45, Lys-127, and Cys-162 were the catalytic triad in active center, which were essential for the catalytic activity of *G. intermedia* nitrilase. The recombinant nitrilase showed good nitrile conversion efficiency and wide substrate spectrum. This study advances our understanding for the role of fungal nitrilase in nitrile hydrolysis. It is expected that this fungal nitrilase will be a potential candidate for industrial applications in carboxylic acids production.

## Materials and Methods

### Strains, plasmids and culture conditions

The fungal strain of *G. intermedia* CA3-1 was isolated and identified in our laboratory and deposited in the China General Microbiological Culture Collection Center (Beijing, China) with an accession number of CGMCC 4903. *E. coli* DH5α and Rosetta-gami (DE3) were used as the host cells for gene cloning and expression, respectively. pMD19-T vector for cloning of PCR products was purchased from Takara (Japan). Expression vector pET-28a(+) was obtained from Novagen (USA), carrying an N-terminal and a C-terminal His_6_-Tag sequence. All other reagents were of analytical grade and purchased from commercial sources.

*E. coli* and recombinant *E. coli* strains were grown in Luria-Bertani (LB) (yeast extract 5 g/L, tryptone 10 g/L, NaCl 10 g/L) liquid medium at 37°C and 220 rpm. Cultivation of strains harboring plasmid pMD19-T and pET-28a(+) were performed with ampicillin (100 µg/mL) and kanamycin (10 µg/mL), respectively. Furthermore, the culture of *E. coli* Rosetta-gami (DE3) should be additionally added with chloramphenicol (25 µg/mL).

### Cloning and expression procedures

Total RNA was extracted from the mycelia of *G. intermedia* CA3-1 according to the instruction of Trizol Kit (Sangon Biotechnology, Shanghai, China). Analytical results of the extracted total RNA showed that the ratio of A_260_ to A_280_ was 1.95 and the 18S and 28S rRNA bands were clear through the electrophoresis results. It indicated that the total RNA possessed high purity and could be used for cDNA amplification by RT-PCR. Two degenerated primers (forward primer), am and bm derived from multiple sequence alignment of the amino acid sequences of fungal nitrilase protein were designed to amplify the partial cDNA of the nitrilase gene through nested-PCR (Table 2). The reverse primer, M13 Primer M4 was provided by the RNA PCR Kit (AMV) Ver.3.0 (Takara, Japan). For cloning of the partial cDNA sequence using degenerated primers, the total RNA was firstly reverse transcribed with AMV reverse transcriptase, which was performed according to the instructions provided by the manufacturers. And then the cDNA sequence encoding the mature peptide of nitrilase was amplified with forward primer am and reverse primer M13 Primer M4. Further nested-PCR was carried out with forward primer bm and reverse primer M13 Primer M4 to verify the accuracy of the amplification described above.

**Table 2:** All the relevant primer sequences in RT-PCR and OE-PCR.

Primer name	Primer sequence (5′→3′)
P1	CCGGAATTCATGTCCAAGACTCTCAAAGTCG
P2	CCCAAGCTTTCACAGGTCGTTGGCAAAG
M13 Primer M4	GTTTTCCCAGTCACGAC
am	ATGTCCAAGWCYCTCAARGT
bm	GATGGACCAGATCCGAGC
FM45	TATCCAGGAATGAAGACTTGAGGGAAGC
PM45	ATCGGCTTCCCTCAAGTCTTCATTCCTG
FM127	TGGGCTTGATCCTGCGGCGGTGAAGAACAATAG
PM127	TATTGTTCTTCACCGCCGCAGGATCAAGCCCAC
FM162	TGTGCTCCCAGCCGTTAAGACCAGCAAC
PM162	TCTTAACGGCTGGGAGCACACCCAGACACTTC

In order to amplify the DNA sequence encoding for the fungal nitrilase sequence, forward primer P1 and reverse primer P2 were designed (Table 2). An amplified fragment was purified by agarose gel electrophoresis and cloned into the plasmid of pMD19-T by T/A cloning, designated as pMD19T-Nit and transformed into *E. coli* DH5α competent cells. Finally, the positive clone was sequenced.

The DNA fragment was ligated with the plasmid of pET-28a(+). The resulting recombinant plasmid was designated as pET28a(+)-Nit. The plasmid pET28a(+)-Nit was transformed to *E. coli* host strain Rosetta-gami(DE3) by heat shock, named as *E. coli* Rosetta-gami(DE3)/pET28a(+)-Nit. The positive clones were identified by colony PCR and double digestion. For induction of the recombinant fungal nitrilase, 0.5 mM isopropyl β-D-thiogalactoside (IPTG) was added when OD_600 nm_ of the recombinant strain reached 0.6–0.8 in LB medium containing 10 µg/mL kanamycin and 25 µg/mL chloramphenicol (37°C and 220 rpm). And then, the strain was incubated at 25°C and 220 rpm for another 20 h.

### Sequence analysis

The amplified DNA fragments were sequenced by Sangon Biotechnology (Shanghai, China). The cDNA and DNA sequences of the fungal nitrilase gene were analyzed using the DNAMAN version 5.2.2 software (Lynnon Biosoft, Canada). Amino acid homology comparisons between *Gibberella* nitrilase and other nitrilase gene were performed with the BLAST program in the GenBank Database.

### Overlap extension PCR

The nitrilase variants of *G. intermedia* CA3-1 with mutation at the positions of Glu-45, Lys-127 and Cys-162 were constructed via OE-PCR. The partial fragments on each side of the mutant site were separately amplified with two pair of primers (P1-FM, PM-P2) using Pfu DNA polymerase (Fermentas, Canada). (All the relevant primer sequences are provided in Table 2.) There is some overlap between primers FM and PM. The fragment in proximity to 5′ end was amplified using forward primer P1 as well as reverse primer FM, and the fragment in proximity to 3′ end using forward primer PM as well as reverse primer P2. The two PCR fragments with correct size were purified by agarose gel electrophoresis and mixed adequately. The resulting mixture was subjected to self-extension at 72°C for 10 min and used as the template of another round of PCR with P1–P2 as the primers. The amplified DNA fragment was purified, ligated with pMD19-T vector and transformed into *E. coli* DH5α. This construct was then sequenced. Correct fragments were digested using *Eco*RI and *Hind*III and inserted into pET-28a(+), followed by transformation into Rosetta-gami(DE3). Positive clones were picked out and their nitrilase activities were determined.

### Biotransformation reaction

Enzymatic hydrolysis of 3-cyanopyridine was carried out with shaking at 30°C in a reaction mixture (50 mL) consisting of 100 mM sodium phosphate buffer (pH 7.2), 100 mM 3-cyanopyridine, and 0.375 g resting cells (dry weight). Samples were withdrawn at fixed intervals and the reaction was terminated through centrifugation at 10,000×*g* (Eppendorf 5430R, Germany) for 5 min to remove cells. The experiments were performed for at least three times.

### Nitrilase purification

The cells of recombinant strain were harvested by centrifugation (8000×*g*, 10 min, 4°C) and resuspended in phosphate buffer (100 mM, pH 7.4) with a final concentration of 20 mg dry cell/mL. It was disrupted at 4°C by ultrasonication with the ultrasonic cell disruptor (Scientz Biotechnology, JY92-II, Ningbo, China) which was operated at 200 W for 300 cycles (working 3 s and intervals 7 s as 1 cycle). The cell debris was removed by centrifugation (14,000×*g*, 30 min, 4°C). The cell-free extracts were applied onto a Ni-NTA superflow column (10 mL), which was equilibrated with a binding buffer (50 mM NaH_2_PO_4_-Na_2_HPO_4_, pH 7.4; 500 mM NaCl). Unbound proteins were washed out from the column with a washing buffer (50 mM NaH_2_PO_4_-Na_2_HPO_4_, pH 7.4; 500 mM NaCl; 300 mM imidazole). And then the elution was carried out with a washing buffer (50 mM NaH_2_PO_4_-Na_2_HPO_4_, pH 7.4; 500 mM NaCl; 400 mM imidazole). The target protein was analyzed by SDS-PAGE and used for further experiments or stored at −80°C. Protein concentration was determined by the Coomassie brilliant blue G-250 dye-binding method of Bradford [33] with bovine serum albumin as the standard.

### Nitrilase assay

The standard assays were carried out by mixing the substrate 3-cyanopyridine (50 mM) and the enzyme in sodium phosphate buffer (100 mM, pH 7.2). The mixture (1 mL of final volume) reacted at 30°C with shaking at 120 rpm for 10 min after 5-min preincubation in waterbath of 30°C. And the reaction was terminated by the addition of 2 M HCl (0.1 mL). The enzyme activity was determined by measuring the generated ammonia in the reaction mixture [10]. This assay is based on the phenol–hypochlorite method. The ammonia and nicotinic acid can be produced simultaneously from 3-cyanopyridine hydrolysis with the molar ratio of 1∶1. Through the color reaction, blue complexes are generated within 15 min at 27°C, which could remain stable for at least 24 hours. Its optical density can be determined at 630 nm. One unit of the enzyme activity was defined as the amount of enzyme producing 1 µmol of ammonia per minute under the conditions described above. All assays were performed in triplicate.

The optimal pH and temperature for nitrilase reaction was determined as described above but using different buffer solutions (Sodium citrate buffer, pH 4.0–5.8; sodium phosphate buffer, pH 5.8–8.5; tris-HCl buffer, pH 8.5–10.0) or at different temperatures (20–55°C).

The effects of various metal ions and EDTA (final concentration of 1 mM; see Figure 4c) or cosolvents (5% and 20% v/v of methanol, ethanol, 2-propanol, 2-chloride methane, and 3-chloride methane) on nitrilase activity were also estimated.

The substrate spectrum was assayed as described above but using 20 mM various nitriles as the substrate (see Table 1). The conversion was assayed by determining ammonia generated in the reaction mixture as aforementioned. A control experiment with inactivated enzyme as the catalyst was performed for each substrate.

To determine the kinetic parameters of the purified nitrilase for 3-cyanopyridine hydrolysis, the activity assays were performed with different substrate concentrations. Kinetic parameters *V*_max_ and *K*_m_ were calculated through the Hanes-Woolf plot (a plot of [*S*] versus [*S*]/*V*). [*S*] is the substrate concentration used for conversion reaction and *V* is the initial velocity at different substrate concentrations.

### Analytical methods

The dry cell weight was determined after collecting by centrifugation at 8,000×*g* for 10 min and dried at 115°C for 2 h. The optical density of culture broth was spectrophotometrically determined at 600 nm (Mapada UV-1800, Shanghai, China).

The analysis of 3-cyanopyridine, nicotinamide and nicotinic acid was performed by HPLC using a Atlantis dC18 column (5.0 µm, 150×4.6 mm; Waters, USA) at the wavelength of 268 nm, column temperature of 30°C. The mobile phase consisted of 0.01% phosphoric acid and methanol (95∶5) with a flow rate of 0.5 mL/min.
